# Hepatic Fat Content and Liver Enzymes Are Associated with Circulating Free and Protein-Bound Advanced Glycation End Products, Which Are Associated with Low-Grade Inflammation: The CODAM Study

**DOI:** 10.1155/2019/6289831

**Published:** 2019-05-14

**Authors:** Mitchell Bijnen, Marleen M. J. van Greevenbroek, Carla J. H. van der Kallen, Jean L. Scheijen, Marjo P. H. van de Waarenburg, Coen D. A. Stehouwer, Kristiaan Wouters, Casper G. Schalkwijk

**Affiliations:** ^1^Department of Internal Medicine, MUMC, Maastricht, Netherlands; ^2^CARIM, MUMC, Maastricht, Netherlands

## Abstract

Advanced glycation end products (AGEs) accumulate in fatty livers and may contribute to low-grade inflammation (LGI), potentially via their receptor, RAGE. It is unknown if the AGE accumulation in fatty livers results in elevated circulating AGEs. In a cohort study, we investigated the association of liver fat and hepatocellular damage with circulating AGEs and soluble RAGE (sRAGE) and subsequently the association of circulating AGEs and sRAGE with LGI. Cross-sectional associations of liver fat percentage (eLF%; ln-transformed) and liver enzymes (LE score; standardized) with circulating AGEs (free CML, CEL, and MG-H1 in nM and protein-bound CML, CEL, and pentosidine in nmol/mmol lysine; ln-transformed) and sRAGE (pg/ml, ln-transformed) and additionally of AGEs and sRAGE with LGI (standardized) were determined by multiple linear regression. eLF% was positively associated with circulating free CEL (*β* = 0.090; 95% CI 0.041; 0.139) but inversely with protein-bound CML (*β* = −0.071; 95% CI -0.108; -0.034). Similarly, the LE score was positively associated with free CML (*β* = 0.044; 95% CI 0.012; 0.076) and CEL (*β* = 0.040; 95% CI 0.009; 0.072) but inversely with protein-bound CML (*β* = −0.037; 95% CI -0.060; -0.013). Free CML (*β* = 0.297; 95% CI 0.049; 0.545) was positively associated with LGI, while protein-bound CML (*β* = −0.547; 95% CI -0.888; -0.207) was inversely associated, although this association was absent after adjustment for BMI. eLF% and LE score were not associated with sRAGE and sRAGE not with LGI after adjustment for BMI. Liver fat and enzymes were positively associated with circulating free AGEs, which were associated with LGI. In contrast, inverse relations were observed of liver fat and enzymes with circulating protein-bound AGEs and of protein-bound AGEs with LGI. These data suggest that hepatic steatosis and inflammation affect the formation and degradation of hepatic protein-bound AGEs resulting in elevated circulating free AGE levels. These alterations in AGE levels might influence LGI, but this is likely independent of RAGE.

## 1. Introduction

Nonalcoholic fatty liver disease (NAFLD) is a spectrum of liver abnormalities ranging from steatosis (fatty liver) to nonalcoholic steatohepatitis (NASH), fibrosis, and potentially even cirrhosis. NASH is characterized by both steatosis and inflammation, of which the latter causes hepatocellular injury and over time irreversible liver injury [1]. Moreover, NASH is associated with cardiovascular disease (CVD), seemingly due to hepatic inflammation considering that long-term survival of CVD-related diseases is lower in NASH patients than in NAFLD patients with steatosis only [2, 3]. Therefore, it is highly relevant to investigate the causes of hepatic inflammation and how these might affect CVD risk.

Fat accumulation in the liver, i.e., steatosis, can cause oxidative stress, increased lipid peroxidation, and release of inflammatory cytokines [4, 5]. Higher levels of oxidative stress and lipid peroxidation, accompanied by inflammation-induced elevated metabolic rate, stimulate the formation of advanced glycation end products (AGEs) [6, 7]. These sugar-modified proteins are capable of disturbing intracellular protein function, cross-linking extracellular matrix (ECM) proteins, and activating the receptor of advanced glycation end products, RAGE [8]. AGEs can be present in both the free (glycated free amino acids) and protein-bound (glycated amino acids within a protein) form. Considering that many of the amino acids in the circulation are derived from degraded proteins, free AGEs are likely derived from degradation of protein-bound AGEs [9]. Major AGEs include N*^ε^*-(carboxymethyl)lysine (CML), N*^ε^*-(1-carboxyethyl)lysine (CEL), N*^δ^*-(5-hydro-5-methyl-4-imidazolon-2-yl)-ornithine (MG-H1), and pentosidine. Binding and stimulation of the receptor for advanced glycation end products (RAGE) by protein-bound CML (PB-CML), protein-bound CEL (PB-CEL), and MG-H1 can increase oxidative stress and upregulate transcription of NF-*κ*B-dependent genes, i.e., inflammatory genes [10–12]. Furthermore, our group demonstrated that RAGE can trap the protein-bound form of AGEs in obese adipose tissue thereby inducing adipose tissue inflammation [13]. Besides as a cell membrane-bound form that stimulates inflammation, RAGE can also be present as soluble RAGE (sRAGE) in plasma due to alternative splicing (i.e., esRAGE) or cleavage of the already cell-bound form [14, 15]. The functional role of sRAGE has not yet been fully elucidated, but it is thought to act as a decoy receptor for its ligands [15]. Alternatively, it might simply be an indication of RAGE activity as it has been associated with CVD and diabetes [16, 17].

We previously showed that CML accumulates in human fatty livers. This accumulation was more pronounced in livers with more severe steatosis and inflammation [6]. In addition, we demonstrated that PB-CML exerts proinflammatory effects on hepatocytes via RAGE, resulting in the production of inflammatory cytokines [6]. We now hypothesize that AGEs that accumulate in the fatty liver are released into the circulation and also activate RAGE in the liver, thereby contributing to low-grade inflammation (LGI). In a human cohort study, we investigated the association of (1) liver fat and liver enzymes with circulating AGEs or sRAGE and (2) the association of circulating AGEs and sRAGE with LGI.

## 2. Materials and Methods

### 2.1. Subjects and Study Design

Cohort on Diabetes and Atherosclerosis Maastricht (CODAM) is a cohort study of 574 subjects, selected based on a moderately increased risk of cardiometabolic disease from a large cohort in the general population as previously described [18]. The current cross-sectional analyses were performed in 505 subjects as subjects with missing data (either main dependent (outcome) or main independent variable or important covariate) were excluded. The study protocol was approved by the Medical Ethical Committee of the Maastricht University Medical Centre, in line with the ethical guidelines of the 1975 Declaration of Helsinki, and all subjects gave written informed consent.

### 2.2. Liver Fat Estimation

Liver fat percentage (eLF%) was estimated using a magnetic resonance spectroscopy- (MRS-) validated equation developed by Kotronen et al. [19]. Briefly, liver fat content is estimated based on liver enzymes (ASAT (aspartate amino transferase) and ASAT/alanine transaminase (ALAT) ratio), fasting insulin levels, presence of the metabolic syndrome, and presence of type 2 diabetes. In addition, the fatty liver index (FLI) as described by Bedogni et al. was used. This estimation is based on an algorithm using BMI, waist circumference, triglycerides, and gamma-glutamyl transferase (GGT) levels and was validated using ultrasound and MRS [20, 21].

### 2.3. Liver Enzymes and Inflammatory Markers

ASAT, ALAT, and GGT were measured in EDTA plasma as previously described [22]. Plasma markers of low-grade inflammation (IL-6, IL-8, TNF-*α*, CRP, SAA, and sICAM1) were measured using an MSD multiplex assay.

### 2.4. Measurement of Advanced Glycation End Products

Free CML, CEL, and MG-H1 and PB-CML, PB-CEL, and protein-bound pentosidine (PB-pentosidine) were analysed in EDTA plasma by ultraperformance liquid chromatography tandem mass spectrometry (UPLC MS/MS). Details of the measurement of plasma AGEs have been previously described [23].

### 2.5. Measurement of sRAGE

sRAGE (esRAGE and shed forms of RAGE) levels were determined in EDTA plasma using a human RAGE quantikine ELISA (R&D Systems) according to the manufacturer's instructions. Intra- and interassay coefficients of variation were 7.6 and 2.6%, respectively.

### 2.6. Other Covariates

Body mass index (BMI), smoking status, mean alcohol consumption, medication use, prior CVD, and glomerular filtration rate (eGFR; estimated using the short Modification of Diet in Renal Disease equation) were determined as previously reported [22].

### 2.7. Statistical Analysis

Study population characteristics were compared across tertiles of liver fat percentage using standard one-way ANOVA with a Bonferroni post hoc test for normally distributed variables, Kruskal-Wallis one-way ANOVA with a Bonferroni post hoc test for non-normally distributed variables, and a Chi-square test with a Bonferroni correction for categorical variables. A combined liver enzyme (LE) or low-grade inflammation (LGI) score was calculated as previously described [22]. In brief, all LEs or LGI plasma markers were ln-transformed to obtain a normal distribution. For each individual, these ln-transformed values were transformed into *Z*-scores (i.e., standardized values). Next, the three standardized LEs and six inflammation markers, respectively, were averaged to obtain a composite score, which was again standardized. The resulting LE score and LGI score were used in subsequent analyses. All AGEs, eLF%, and sRAGE were also ln-transformed prior to analyses. FLI was standardized before analyses.

The cross-sectional associations of liver fat and liver enzymes (main independent variables) with AGEs and sRAGE (outcomes) and additionally of AGEs and sRAGE (main independent variables) with LGI (outcome) were examined with the use of multiple linear regression analyses with adjustments for the following potential confounders: age (years), sex (male/female), alcohol consumption (g/day), current smoker (Y/N), prevalent CVD (Y/N), use of medication (glucose-lowering (Y/N), lipid-modifying (Y/N), and antihypertensive (Y/N)), eGFR (ml/min/1.73 m^2^), and BMI (kg/m^2^). All linear regression analyses were performed with four different models as follows: model 1: crude model; model 2: adjustment for age and sex; model 3: adjustment for age, sex, alcohol, smoking, CVD, medication, and eGFR; model 4: adjustment for age, sex, alcohol, smoking, CVD, medication, eGFR, and BMI. All statistical analyses were performed using the SPSS for Windows, version 25.0 (IBM Corp.), and all data were considered statistically significant at *P* ≤ 0.05.

## 3. Results

### 3.1. Study Population

In Table 1, the study population is presented according to tertiles of eLF%. Subjects with more severe steatosis in general had a higher prevalence of type 2 diabetes and CVD and accordingly used more medication. Moreover, individuals in the highest liver fat tertile had the highest BMI, fasting glucose, triglyceride, and HbA1c levels but the lowest HDL level. These worse metabolic characteristics were accompanied by higher levels of low-grade inflammation markers and liver enzymes. PB-pentosidine, PB-CML, and sRAGE were lower, while free CEL was higher in those with the highest amount of liver fat.

### 3.2. Association of Liver Fat with Free and Protein-Bound AGEs and sRAGE

In linear regression analyses, eLF% was positively associated with free CML (*β* = 0.042; 95% CI 0.002; 0.082) and CEL (*β* = 0.096; 95% CI 0.058; 0.135) but inversely with PB-pentosidine (*β* = −0.059; 95% CI -0.1; -0.018), PB-CML (*β* = −0.115; 95% CI -0.143; -0.087), and sRAGE (*β* = −0.097; 95% CI -0.146; -0.048). Adjustment for age, sex, alcohol consumption, smoking, prior CVD, medication use, and eGFR (model 3) mainly affected the positive association of eLF% with free CML, which was no longer significant (*β* = 0.041; 95% CI -0.002; 0.084). After additional adjustment for BMI (model 4), eLF% was still positively associated with free CEL and inversely with PB-CML, but the association with free CML, PB-pentosidine, or sRAGE was no longer significant. eLF% was not associated with PB-CEL or MG-H1 (Table 2). Additional analyses were performed with FLI instead of the eLF% as a measure of liver fat and yielded comparable results (Suppl.  Table 1). However, the positive association between FLI and free CML now remained significant after adjustment of several confounders (model 3), and the inverse association between FLI and PB-pentosidine was also retained after adjustment for BMI (model 4). Of note, adjusting for waist circumference instead of BMI in model 4 did not materially change the results (data not shown).

### 3.3. Association of Liver Enzymes with Free and Protein-Bound AGEs and sRAGE

Next, we investigated the association of the LE score (as a measure of hepatocellular injury) with AGEs and sRAGE (Table 2). The LE score was positively associated with free CML (*β* = 0.030; 95% CI 0; 0.060) and free CEL (*β* = 0.047; 95% CI 0.018; 0.077), but an inverse association between the LE score and PB-CML (*β* = −0.061; 95% CI -0.083; -0.039) or the LE score and sRAGE (*β* = −0.072; 95% CI -0.109; -0.034) was observed. The positive associations with free CML (*β* = 0.046; 95% CI 0.015; 0.077) and free CEL (*β* = 0.049; 95% CI 0.019; 0.079) were stronger after additional adjustment (model 3), while the association with PB-CML (*β* = −0.054; 95% CI -0.077; -0.031) was slightly weaker and the association with sRAGE was no longer significant (*β* = −0.027; 95% CI -0.067; 0.012). The LE score was not associated with PB-CEL or MG-H1. Neither adjustment for BMI (Table 2) nor for waist circumference (data not shown) altered these associations substantially.

### 3.4. Association of Liver Fat and Liver Enzymes with LGI

In our cohort, we next investigated the associations between liver fat or liver enzymes and the LGI score (Table 3). There was a strong association of eLF% (*β* = 0.494; 95% CI 0.387; 0.601) and the LE score (*β* = 0.310; 95% CI 0.226; 0.393) with LGI. Additional adjustment did not substantially affect these associations. Moreover, when employing the FLI instead of eLF% (Suppl.  Table 2) or waist circumference instead of BMI (data not shown), the results were highly comparable.

### 3.5. Association of Free and Protein-Bound AGEs and sRAGE with LGI

AGEs can trigger inflammation via RAGE, potentially contributing to LGI and could thereby explain, at least in part, the association of liver fat and liver enzymes with LGI. Therefore, we investigated the association of AGEs and sRAGE with the LGI score. Free CML (*β* = 0.443; 95% CI 0.194; 0.692) and free CEL (*β* = 0.359; 95% CI 0.103; 0.616) were positively associated with LGI, while PB-CML (*β* = −0.631; 95% CI -0.965; -0.298) was inversely associated with this estimate of systemic inflammation. After adjustment for age, sex, alcohol consumption, smoking, prior CVD, medication use, and eGFR (model 3), the positive association between free CEL and LGI was borderline nonsignificant (*β* = 0.259; 95% CI -0.006; 0.525), while the associations of free CML (positive) and PB-CML (inverse) with LGI remained significant. Adjustment for BMI (model 4) strongly attenuated the inverse association between PB-CML and LGI. This association was no longer significant (*β* = −0.251; 95% CI -0.592; 0.090), and only the positive association between free CML and LGI remained (Table 4). To examine if free CML indeed contributes to the above-described strong association of eLF% or LE score with LGI, we performed an additional analysis in which we adjusted that association for free CML. Adjustment for free CML reduced the association between eLF% and LGI slightly from *β* = 0.429 (95% CI 0.290; 0.568) to *β* = 0.420 (95% CI 0.281; 0.558) and the association between the LE score and LGI from *β* = 0.292 (95% CI 0.205; 0.380) to *β* = 0.283 (95% CI 0.195; 0.371). No association was observed between sRAGE, PB-CEL, PB-pentosidine or MG-H1, and LGI (Table 4). Performing these analyses with adjustment for waist circumference instead of BMI in model 4 did not alter the associations (data not shown).

## 4. Discussion

The present study revealed that liver fat and liver enzymes were positively associated with circulating free AGEs, while inverse associations were observed with circulating protein-bound AGEs. In addition, free AGEs were positively associated with LGI, but an inverse association was determined between protein-bound AGEs and LGI, albeit this latter association was explained by BMI. Lastly, there was no association observed of liver fat and liver enzymes with sRAGE or between sRAGE and LGI after adjustment for BMI.

We demonstrated that a higher hepatic fat content, a hallmark of NAFLD, is associated with AGEs and sRAGE, but a key role is played by obesity and adipose tissue as adjustment for BMI attenuated these associations. However, even after adjustment for this potential confounder, a positive association between liver fat and free AGEs (only CEL and not CML) and an inverse association between liver fat and protein-bound AGEs (pentosidine and PB-CML) was still observed. Lipid peroxidation contributes to CEL formation making its generation likely in metabolically active and lipid-rich environments such as the fatty liver [7]. Indeed, levels of other lipid peroxidation products (e.g. malondialdehyde and 8-isoprostane) are elevated in the circulation of NAFLD patients [24, 25]. Moreover, hepatic steatosis was shown to lead to lipid peroxidation in mice [26]. In line with our data, Palma-Duran et al. recently reported elevated levels of circulating CEL and not CML in NAFLD patients in a case-control study [27].

The inverse relationship between hepatic steatosis and circulating protein-bound AGEs might suggest either trapping of AGEs in the liver or an elevated breakdown of (modified) proteins in fatty livers. We previously reported trapping of PB-CML in adipose tissue of obese mice and reduced levels of circulating PB-CML in obese subjects [13]. In the liver, we also observed trapping of CML-modified albumin, although the amount of trapped PB-CML in the liver was not compared between healthy and steatotic livers [13]. Therefore, it is possible that a higher amount of PB-CML is trapped in fatty livers, which could explain the inverse direction of the association between liver fat and circulating PB-CML. Another explanation could be alterations in protein metabolism, specifically an enhanced protein breakdown, in NAFLD. A recent study indeed reported elevated levels of plasma amino acids, including leucine, a marker of protein breakdown, in NAFLD patients [28, 29]. This could also explain the inverse relationship between liver fat and pentosidine. Pentosidine is formed through the slow Maillard reaction and therefore mainly affects long-living proteins, which are typically ECM components such as collagen [30]. In contrast to enhanced protein breakdown, Munsterman et al. reported inhibition of hepatic ECM degradation by hepatic stellate cells in NAFLD [31]. Reduced ECM degradation could explain the observed lower circulating pentosidine levels as less modified proteins are released from the ECM, albeit Munsterman et al. reported this in a more advanced stage of NAFLD [31].

We found a strong inverse association between liver fat and sRAGE, but this association was largely dependent on BMI. Several studies have shown that obesity is a strong determinant of sRAGE levels explaining why the association is no longer present after adjustment for this confounder [32, 33]. In accordance with our data, Palma-Duran et al. reported a decrease of sRAGE in NAFLD patients [27]. Interestingly, this difference between cases and controls in their study was observed despite matching for BMI. However, their study used diagnosed NAFLD patients in whom the liver status might be worse than our population. Possibly, sRAGE levels are influenced more by the liver and independently from the obese adipose tissue in patients with worse NALFD.

Our study implies that hepatic injury, represented by liver enzyme levels, is associated with higher levels of circulating free AGEs (CML and CEL), but lower levels of protein-bound AGEs (PB-CML). These findings are in agreement with our previous study revealing elevated levels of CML in livers with more severe steatosis and inflammation, which causes liver injury [6]. Furthermore, hepatic inflammation is accompanied by oxidative stress given that hepatic immune cells are important producers of reactive oxygen species (ROS) [34]. High oxidative stress causes lipid peroxidation and contributes to AGE formation, mainly CML and CEL [7, 35].

The association of hepatocellular injury with elevated circulating free CML, but lower circulating PB-CML, suggests enhanced breakdown of proteins by liver injury and the accompanying hepatic inflammation. As previously described, protein degradation is enhanced in NAFLD patients [29]. Moreover, circulating levels of free amino acids correlated strongly with liver enzymes and even hepatic injury and inflammation [29]. These findings make it likely that more intrahepatic PB-CML is degraded to circulating free CML in subjects with more hepatocellular injury.

Our results also show an association of liver fat and liver injury with LGI corroborating that liver disease might contribute to elevated inflammatory cytokines and thereby maintenance of low-grade inflammation [36, 37].

The observed positive association of free CML and CEL with LGI suggests that these plasma-free AGEs might influence LGI. However, the association of CEL with LGI disappeared after adjustment for BMI implying that this association depends more on adiposity and the adipose tissue inflammation that accompanies it. Free CML remained associated with LGI even after adjustment for BMI. However, the very modest effect of additional adjustment for free CML suggested that the strong association of hepatic steatosis and injury with LGI was not mediated by circulating free CML levels. Indeed, it has been described that free CML is unable to stimulate RAGE and cause inflammation [11]. Therefore, any effects on LGI by free CML are likely to be indirect or due to disturbance of intracellular function and not via RAGE [8].

In line with this notion, we observed that sRAGE was not associated with LGI despite its potential role as a decoy receptor for its ligands or as indicator of AGE-RAGE axis activity [15–17]. In contrast, Palma-Duran et al. did report an inverse correlation between sRAGE and either TNF-*α* and CRP, two components of our LGI score [27]. This discrepancy could be explained by the other markers used to calculate our LGI score as these markers might not correlate well with sRAGE. Indeed, when performing a similar analysis, we observed a significant inverse correlation between sRAGE and CRP as well, but the correlation between sRAGE and TNF-*α* was not present (data not shown). However, we believe that our LGI score is a representative measure of low-grade inflammation and is preferable over a few single markers. Another explanation for discrepancies can be found in the difference in severity of liver disease between the studies. As previously mentioned, Palma-Duran et al. used diagnosed NAFLD patients in comparison to our population without diagnosis of NAFLD [27]. Interestingly, the inverse association between PB-CML and LGI also disappeared after adjustment for BMI. Likely, this has to do with the aforementioned trapping of PB-CML in the adipose tissue [13].

Of note, AGEs can form exogenously and are present in many food products [38]. The intestines absorb these dietary AGEs and we have recently demonstrated that a higher dietary AGE intake is associated with higher levels of free AGEs in plasma and urine [39]. These data support the idea that circulating free AGE levels are influenced by many factors including dietary AGE intake and as reported in the present study, liver health status.

Our study has some limitations. Given our cross-sectional study design, we cannot draw causal conclusions on the investigated relationships. Prospective studies are required to better understand the contribution of liver disease to AGE formation and subsequently circulating AGEs and low-grade inflammation. Another important consideration is the estimation of liver fat employed in the study. We used two different equations to estimate liver fat content but did not measure liver fat content using imaging techniques or quantify hepatic steatosis or inflammation in liver biopsies. These procedures were not feasible in our large human cohort due to cost or ethical concerns. However, the two measures of liver fat (eLF% and FLI) used in this study have been validated in previous studies and revealed similar associations in the present study [19, 20].

## 5. Conclusions

Our findings suggest an elevated formation of AGEs in fatty and injured livers accompanied with an enhanced breakdown of the formed protein-bound AGEs resulting in altered levels of circulating AGEs. Potentially, circulating AGEs can contribute to LGI.

## Figures and Tables

**Table 1:** General characteristics of the study population (*n* = 505) according to tertiles of eLF%.

	All participants	Liver fat according to tertiles of eLF%			*P* value
	(*n* = 505)	Lowest (*n* = 168)	Middle (*n* = 169)	Highest (*n* = 168)	
Age (years)	59.5 ± 7.0	58.5 ± 7.3	59.8 ± 7.4	60.3 ± 6.3	0.057
Male sex (%)	62.4	54.2	60.9	72^∗∗^	0.003
NGM/IGM/T2DM (%)	53.5/22.6/24.0	83.3/15.5/1.2	56.8/30.2/13.0^∗∗∗^	20.2/22.0/57.7^∗∗∗^^###^	<0.001
CVD (%)	26.9	19.6	23.7	37.5^∗∗∗^^#^	0.001
Current smoker (%)	20.8	22.6	21.9	17.9	0.853
Alcohol (g/day)	8.6 (1.3–22.8)	8.5 (2.4–22.7)	9.9 (1.4–24.2)	7.1 (0.5–21.2)	0.311
Antihypertensive medication (%)	37.4	23.2	35.5^∗^	53.6^∗∗∗^^##^	<0.001
Lipid-modifying medication (%)	18.2	12.5	16.6	25.6^∗∗^	0.006
Glucose-lowering medication (%)	12.1	0.6	5.3^∗^	30.4^∗∗∗^^###^	<0.001
Body mass index (kg/m²)	28.4 ± 4.2	25.8 ± 3.1	28.4 ± 3.4^∗∗∗^	31.0 ± 4.4^∗∗∗^^###^	<0.001
Waist circumference (cm)	98.9 ± 11.9	90.3 ± 9.0	99.1 ± 9.2^∗∗∗^	107.4 ± 10.6^∗∗∗^^###^	<0.001
Fasting glucose (mM)	5.58 (5.20–6.34)	5.20 (4.94–5.44)	5.57 (5.22–6.00)^∗∗∗^	6.57 (5.84–7.62)^∗∗∗^^###^	<0.001
Triglycerides (mM)	1.4 (1.0–1.9)	1.0 (0.8–1.4)	1.5 (1.1–2.0)^∗∗∗^	1.8 (1.4–2.2)^∗∗∗^^###^	<0.001
Cholesterol (mM)	5.21 ± 0.93	5.14 ± 0.90	5.31 ± 0.94	5.17 ± 0.95	0.197
LDL cholesterol (mM)	3.32 ± 0.87	3.24 ± 0.86	3.43 ± 0.88	3.29 ± 0.87	0.120
HDL cholesterol (mM)	1.20 ± 0.35	1.41 ± 0.35	1.16 ± 0.32^∗∗∗^	1.03 ± 0.25^∗∗∗^^###^	<0.001
HbA1c (%)	5.80 (5.50–6.20)	5.60 (5.30–5.80)	5.80 (5.50–6.10)^∗∗∗^	6.20 (5.80–6.90)^∗∗∗^^###^	<0.001
HOMA2-IR	1.58 (1.09–2.51)	1.02 (0.80–1.26)	1.56 (1.21–2.00)^∗∗∗^	3.21 (2.37–4.55)^∗∗∗^^###^	<0.001
IL-6 (pg/ml)	1.56 (1.12–2.28)	1.25 (0.96–2.18)	1.60 (1.17–2.20)^∗^	1.80 (1.33–2.57)^∗∗∗^	<0.001
IL-8 (pg/ml)	4.34 (3.58–5.53)	4.18 (3.44–5.23)	4.30 (3.50–5.13)	4.78 (3.83–6.30)^∗∗∗^^##^	<0.001
TNF-*α* (pg/ml)	6.25 (5.23–7.61)	5.94 (5.02–7.01)	6.34 (5.30–7.51)	6.60 (5.43–7.96)^∗∗∗^	0.002
CRP (mg/l)	2.04 (0.92–3.97)	1.07 (0.59–2.72)	2.20 (1.20–4.33)^∗∗∗^	2.71 (1.46–5.00)^∗∗∗^	<0.001
SAA (mg/l)	1.42 (0.98–2.27)	1.20 (0.87–2.16)	1.52 (1.02–2.41)^∗^	1.52 (1.05–2.33)^∗∗^	0.004
sICAM-1 (ng/ml)	212.5 (186.8–242.7)	195.4 (177.8–221.5)	211.0 (189.1–236.5)^∗∗∗^	231.8 (204.9–257.8)^∗∗∗^^###^	<0.001
Low-grade inflammation score	0.00 ± 1.00	−0.42 ± 0.97	0.03 ± 0.89^∗∗∗^	0.38 ± 0.98^∗∗∗^^##^	<0.001
PB-pentosidine (nmol/mmol lysine)	0.43 (0.36–0.53)	0.46 (0.39–0.55)	0.44 (0.36–0.52)	0.41 (0.35–0.50)^∗^	0.015
PB-CML (nmol/mmol lysine)	34.6 (29.6–41.0)	37.1 (32.3–44.9)	35.2 (31.1–40.6)	31.1 (26.4–38.0)^∗∗∗^^###^	<0.001
PB-CEL (nmol/mmol lysine)	23.3 (19.0–29.2)	22.8 (19.5–26.9)	24.2 (19.0–30.3)	22.9 (18.6–29.5)	0.250
Free CML (nM)	79.5 (61.2–98.6)	76.0 (60.3–92.9)	80.4 (61.0–100.1)	82.2 (64.1–102.5)	0.144
Free CEL (nM)	45.5 (37.0–58.0)	42.7 (34.8–52.4)	45.9 (38.7–56.7)	51.0 (38.2–63.0)^∗∗∗^	<0.001
Free MG-H1 (nM)	123.8 (87.5–176.6)	127.2 (90.8–168.1)	121.0 (85.6–172.5)	119.6 (85.0–198.3)	0.810
sRAGE (pg/ml)	1250 (893–1604)	1402 (1112–1756)	1229 (838–1567)^∗∗^	1155 (850–1440)^∗∗∗^	<0.001
ALAT (U/l)	22.2 (17.2–27.9)	17.1 (14.3–21.2)	22.4 (18.0–26.7)^∗∗∗^	28.6 (23.2–36.3)^∗∗∗^^###^	<0.001
ASAT (U/l)	19.8 (16.5–24.2)	18.2 (14.7–21.4)	19.3 (16.6–23.3)^∗∗^	22.7 (19.0–27.6)^∗∗∗^^###^	<0.001
GGT (U/l)	24.0 (17.0–37.0)	18.0 (13.0–23.8)	26.0 (18.0–37.5)^∗∗∗^	34.0 (24.0–48.8)^∗∗∗^^###^	<0.001
Liver enzyme score	0.00 ± 1.00	−0.64 ± 0.72	−0.02 ± 0.81^∗∗∗^	0.66 ± 1.00^∗∗∗^^###^	<0.001
eLF% (%)	4.79 (2.35–8.62)	2.11 (1.79–2.35)	4.79 (3.80–5.89)^∗∗∗^	10.64 (8.60–14.53)^∗∗∗^^###^	<0.001
FLI	55.7 ± 27.8	30.6 ± 19.3	57.8 ± 22.4^∗∗∗^	78.7 ± 16.8^∗∗∗^^###^	<0.001
eGFR (ml/min/1.73 m^2^)	91.5 ± 18.6	89.3 ± 15.1	90.8 ± 17.9	94.5 ± 21.9	0.028

Data are expressed as mean ± SD, median (interquartile range) or percentages. The minimum and maximum of eLF% tertiles were (0.85–2.91), (2.92–6.97), and (6.98–36.65) %, respectively. eLF%: estimated liver fat %; NGM: normal glucose metabolism; IGM: impaired glucose metabolism; T2DM: type 2 diabetes mellitus; CVD: cardiovascular disease; LDL: low-density lipoprotein; HDL: high-density lipoprotein; HbA1c: hemoglobin A1c; HOMA2-IR: homeostasis model assessment insulin resistance; IL; interleukin; TNF-*α*: tumour necrosis factor-*α*; CRP: C-reactive protein; SAA: serum amyloid A; sICAM1: soluble intercellular adhesion molecule 1; ALAT: alanine aminotransferase; ASAT: aspartate amino transferase; GGT: *γ*-glutamyl transferase; FLI: fatty liver index; eGFR: estimated glomerular filtration rate. ^∗^ *P* < 0.05, ^∗∗^ *P* < 0.01, ^∗∗∗^ *P* < 0.001 vs. lowest tertile. ^#^ *P* < 0.05, ^##^ *P* < 0.01, ^###^ *P* < 0.001 vs. middle tertile.

**Table 2:** Cross-sectional associations of liver fat and liver enzymes with free and protein-bound AGEs and sRAGE.

Outcome	Model	eLF% (%)				Liver enzyme score			
		*β*	95% CI		*P* value	*β*	95% CI		*P* value
Free CML (nM)	1	0.042	0.002	0.082	**0.038**	0.030	0.000	0.060	0.052
	2	0.031	-0.008	0.070	0.122	0.030	-0.001	0.061	0.055
	3	0.041	-0.002	0.084	0.061	0.046	0.015	0.077	**0.004**
	4	0.037	-0.014	0.088	0.151	0.044	0.012	0.076	**0.008**

Free CEL (nM)	1	0.096	0.058	0.135	**<0.001**	0.047	0.018	0.077	**0.002**
	2	0.083	0.045	0.121	**<0.001**	0.037	0.007	0.067	**0.016**
	3	0.095	0.054	0.136	**<0.001**	0.049	0.019	0.079	**0.001**
	4	0.090	0.041	0.139	**<0.001**	0.040	0.009	0.072	**0.012**

Free MG-H1 (nM)	1	0.043	-0.019	0.105	0.170	-0.009	-0.056	0.038	0.719
	2	0.023	-0.038	0.084	0.464	-0.019	-0.067	0.028	0.423
	3	0.024	-0.042	0.090	0.481	0.006	-0.042	0.053	0.820
	4	0.036	-0.042	0.115	0.367	0.007	-0.043	0.057	0.789

PB-pentosidine (nmol/mmol lysine)	1	-0.059	-0.100	-0.018	**0.005**	-0.015	-0.047	0.016	0.342
	2	-0.076	-0.117	-0.035	**<0.001**	-0.028	-0.060	0.004	0.088
	3	-0.102	-0.146	-0.058	**<0.001**	-0.032	-0.064	0.001	0.055
	4	-0.051	-0.102	0.001	0.055	-0.007	-0.040	0.026	0.676

PB-CML (nmol/mmol lysine)	1	-0.115	-0.143	-0.087	**<0.001**	-0.061	-0.083	-0.039	**<0.001**
	2	-0.122	-0.150	-0.093	**<0.001**	-0.067	-0.090	-0.044	**<0.001**
	3	-0.105	-0.136	-0.074	**<0.001**	-0.054	-0.077	-0.031	**<0.001**
	4	-0.071	-0.108	-0.034	**<0.001**	-0.037	-0.060	-0.013	**0.002**

PB-CEL (nmol/mmol lysine)	1	0.000	-0.036	0.036	0.992	0.003	-0.025	0.031	0.828
	2	-0.002	-0.039	0.035	0.911	-0.002	-0.030	0.027	0.919
	3	0.013	-0.028	0.055	0.527	0.007	-0.023	0.037	0.652
	4	-0.003	-0.052	0.047	0.920	0.001	-0.030	0.033	0.939

sRAGE (pg/ml)	1	-0.097	-0.146	-0.048	**<0.001**	-0.072	-0.109	-0.034	**<0.001**
	2	-0.076	-0.125	-0.028	**0.002**	-0.040	-0.078	-0.002	**0.039**
	3	-0.076	-0.130	-0.023	**0.005**	-0.027	-0.067	0.012	0.169
	4	-0.039	-0.102	0.025	0.232	-0.010	-0.050	0.031	0.642

*β*-Values are unstandardized regression coefficients and represent the change in AGEs and sRAGE (all ln-transformed) per one unit increase in eLF% (ln-transformed) or the LE score. Model 1: crude model. Model 2: model 1 + adjustment for age and sex. Model 3: model 2 + adjustment for alcohol, smoking, CVD, medication, and eGFR. Model 4: model 3 + adjustment for BMI. 95% CI: 95% confidence interval.

**Table 3:** Associations of liver fat and liver enzymes with low-grade inflammation.

Outcome	Model	eLF% (%)				Liver enzyme score			
		*β*	95% CI		*P* value	*β*	95% CI		*P* value
LGI	1	0.494	0.387	0.601	**<0.001**	0.310	0.226	0.393	**<0.001**
	2	0.493	0.386	0.599	**<0.001**	0.347	0.262	0.431	**<0.001**
	3	0.533	0.415	0.650	**<0.001**	0.356	0.270	0.442	**<0.001**
	4	0.429	0.290	0.568	**<0.001**	0.292	0.205	0.380	**<0.001**

*β*-Values are unstandardized regression coefficients and represent the change in the LGI score per one unit increase in eLF% (ln-transformed) or LE score. Model 1: crude model. Model 2: model 1 + adjustment for age and sex. Model 3: model 2 + adjustment for alcohol, smoking, CVD, medication, and eGFR. Model 4: model 3 + adjustment for BMI. 95% CI: 95% confidence interval.

**(a):** 

Outcome	Model	Free CML (nM)				Free CEL (nM)				Free MG-H1 (nM)			
		*β*	95% CI		*P* value	*β*	95% CI		*P* value	*β*	95% CI		*P* value
LGI	1	0.443	0.194	0.692	**0.001**	0.359	0.103	0.616	**0.006**	0.204	0.014	0.041	0.366
	2	0.322	0.067	0.577	**0.013**	0.282	0.023	0.541	**0.033**	0.128	-0.037	0.293	0.127
	3	0.344	0.086	0.603	**0.009**	0.259	-0.006	0.525	0.056	0.128	-0.041	0.297	0.137
	4	0.297	0.049	0.545	**0.019**	0.152	-0.104	0.409	0.243	0.131	-0.031	0.293	0.112

**(b):** 

Outcome	Model	PB-CML (nmol/mmol lysine)				PB-CEL (nmol/mmol lysine)				PB-pentosidine (nmol/mmol lysine)			
		*β*	95% CI		*P* value	*β*	95% CI		*P* value	*β*	95% CI		*P* value
LGI	1	-0.631	-0.965	-0.298	**<0.001**	-0.047	-0.325	0.231	0.741	-0.059	-0.303	0.185	0.637
	2	-0.704	-1.032	-0.377	**<0.001**	-0.028	-0.302	0.246	0.840	-0.154	-0.398	0.091	0.219
	3	-0.547	-0.888	-0.207	**0.002**	-0.024	-0.293	0.245	0.863	-0.089	-0.338	0.160	0.483
	4	-0.251	-0.592	0.090	0.149	-0.078	-0.335	0.180	0.553	0.120	-0.125	0.365	0.337

**(c):** 

Outcome	Model	sRAGE (pg/ml)			
		*β*	95% CI		*P* value
LGI	1	0.048	-0.154	0.251	0.641
	2	0.019	-0.189	0.226	0.858
	3	0.037	-0.170	0.244	0.725
	4	0.143	-0.057	0.342	0.161

*β*-Values are unstandardized regression coefficients and represent the change in the LGI score per one unit increase in AGEs or sRAGE (all ln-transformed). Model 1: crude model. Model 2: model 1 + adjustment for age and sex. Model 3: model 2 + adjustment for alcohol, smoking, CVD, medication, and eGFR. Model 4: model 3 + adjustment for BMI. 95% CI: 95% confidence interval.

## Data Availability

The datasets generated during and/or analysed during the current study are available from the corresponding author on reasonable request.

## Abbreviations

AGE: Advanced glycation end product
CEL: N*^ε^*-(1-carboxyethyl)lysine
CML: N*^ε^*-(carboxymethyl)lysine
CVD: Cardiovascular disease
LGI: Low-grade inflammation
MG-H1: N*^δ^*-(5-hydro-5-methyl-4-imidazolon-2-yl)-ornithine
NAFLD: Nonalcoholic fatty liver disease
NASH: Nonalcoholic steatohepatitis
RAGE: Receptor for AGEs
TNF: Tumour necrosis factor
UPLC MS/MS: Ultraperformance liquid chromatography tandem mass spectrometry.

## Supplementary Materials

Supplementary Materials Suppl. Table 1: associations of FLI with free and protein-bound AGEs and sRAGE. *β*-Values are unstandardized regression coefficients and represent the change in AGEs and sRAGE (all ln-transformed) per one unit increase in FLI (standardized). Model 1: crude model. Model 2: model 1 + adjustment for age and sex. Model 3: model 2 + adjustment for alcohol, smoking, CVD, medication, and eGFR. Model 4: model 3 + adjustment for BMI. 95% CI: 95% confidence interval; FLI: fatty liver index. Suppl. Table 2: associations of FLI with low-grade inflammation. *β*-Values are unstandardized regression coefficients and represent the change in the LGI score per one unit increase in FLI (standardized). Model 1: crude model. Model 2: model 1 + adjustment for age and sex. Model 3: model 2 + adjustment for alcohol, smoking, CVD, medication, and eGFR. Model 4: model 3 + adjustment for BMI. 95% CI: 95% confidence interval; FLI: fatty liver index.
