# Supplementary material for: Hepatic Fat Content and Liver Enzymes Are Associated with Circulating Free and Protein-Bound Advanced Glycation End Products, Which Are Associated with Low-Grade Inflammation: The CODAM Study
Source: J Diabetes Res. 2019 May 14;2019:6289831. doi: 10.1155/2019/6289831 (PMC6536997; doi:10.1155/2019/6289831)
Supplement: Supplementary Materials — Suppl. Table 1: associations of FLI with free and protein-bound AGEs and sRAGE. β-Values are unstandardized regression coefficients and represent the change in AGEs and sRAGE (all ln-transformed) per one unit increase in FLI (standardized). Model 1: crude model. Model 2: model 1 + adjustment for age and sex. Model 3: model 2 + adjustment for alcohol, smoking, CVD, medication, and eGFR. Model 4: model 3 + adjustment for BMI. 95% CI: 95% confidence interval; FLI: fatty liver index. Suppl. Table 2: associations of FLI with low-grade inflammation. β-Values are unstandardized regression coefficients and represent the change in the LGI score per one unit increase in FLI (standardized). Model 1: crude model. Model 2: model 1 + adjustment for age and sex. Model 3: model 2 + adjustment for alcohol, smoking, CVD, medication, and eGFR. Model 4: model 3 + adjustment for BMI. 95% CI: 95% confidence interval; FLI: fatty liver index. [file 6289831.f1.pdf]

Suppl. table 1. Associations of FLI with free and protein-bound AGEs and sRAGE

		FLI			
Outcome	Model	β	95% CI		P-value
**Free CML**	1	0.031	0.001	0.062	**0.043**
(nM)	2	0.027	-0.004	0.057	0.083
	3	0.033	0.002	0.064	**0.038**
	4	0.048	-0.004	0.099	0.068
**Free CEL**	1	0.064	0.034	0.093	**<0.001**
(nM)	2	0.053	0.023	0.083	**<0.001**
	3	0.058	0.029	0.088	**<0.001**
	4	0.069	0.019	0.119	**0.007**
**Free MG-H1**	1	0.013	-0.034	0.060	0.590
(nM)	2	0.000	-0.047	0.047	0.990
	3	-0.001	-0.048	0.047	0.976
	4	0.004	-0.076	0.083	0.930
**PB-Pentosidine**	1	-0.070	-0.101	-0.039	**<0.001**
(nmol/mmol lysine)	2	-0.086	-0.117	-0.055	**<0.001**
	3	-0.093	-0.124	-0.062	**<0.001**
	4	-0.061	-0.113	-0.009	**0.022**
**PB-CML**	1	-0.089	-0.110	-0.067	**<0.001**
(nmol/mmol lysine)	2	-0.096	-0.117	-0.074	**<0.001**
	3	-0.081	-0.103	-0.058	**<0.001**
	4	-0.063	-0.100	-0.025	**0.001**
**PB-CEL**	1	0.026	-0.002	0.053	0.067
(nmol/mmol lysine)	2	0.024	-0.005	0.003	0.621
	3	0.033	0.003	0.063	**0.031**
	4	0.046	-0.004	0.096	0.069
**sRAGE**	1	-0.090	-0.127	-0.053	**<0.001**
(pg/ml)	2	-0.066	-0.103	-0.029	**0.001**
	3	-0.063	-0.102	-0.025	**0.001**
	4	-0.030	-0.095	0.034	0.354

Suppl. table 2. Associations of FLI with low-grade inflammation

		**FLI**			
Outcome	Model	β	95% CI		P-value
**LGI**	1	0.325	0.242	0.408	**<0.001**
	2	0.343	0.260	0.427	**<0.001**
	3	0.329	0.243	0.416	**<0.001**
	4	0.234	0.090	0.378	**0.002**
